# Testing the Feasibility of Citric Acid-Assisted Nickel Agromining with Tropical and Temperate Hyperaccumulator Plants

**DOI:** 10.1021/acsomega.5c02064

**Published:** 2025-05-22

**Authors:** Luiz Henrique Vieira Lima, Serigne Ndiawar Ly, Raul Santos Rocha de Araújo, Jakson dos Santos Nascimento, Caroline Miranda Biondi, Carlos Alberto Pérez, Renata Santos Rabelo, Guillaume Echevarria, Antony van der Ent, Clístenes Williams Araújo do Nascimento

**Affiliations:** † Department of Agronomy, 67744 Federal Rural University of Pernambuco, Recife 52171-900, Brazil; ‡ Soil and Environment Laboratory, 137665 University of Lorraine, 54000 Nancy, France; § Brazilian Synchrotron Light Laboratory (LNLS), Brazilian Center for Research in Energy and Materials (CNPEM), Campinas, Sao Paulo 13083-970, Brazil; ∥ Econick, 1 Rue Grandville, 54000 Nancy, France; ⊥ Laboratory of Genetics, 4508 Wageningen University and Research, 6708 PW Wageningen, The Netherlands

## Abstract

The efficiency of using chelating agents in combination with hyperaccumulator species for nickel phytoextraction has yielded mixed results thus far. This study evaluated the effects of citric acid application on metal solubility in ultramafic soils and the feasibility of its use for nickel agromining by Berkheya coddii and Bornmuellera emarginata. Four citric acid application doses (0, 20, 40, and 60 mmol kg^–1^) were used in a tropical and in a temperate ultramafic soil for 90 days for B. coddii and 45 days for B. emarginata, and pH and metal solubility assessments were conducted after application. Citric acid application increased the solubility of nickel, cobalt, and manganese in the soil by up to 20-fold but negatively impacted the biomass. Berkheya coddii accumulated up to dozens of times more Ni, Co, Cr, Cu, Mn, and Zn in its leaves than did B. emarginata. However, the citric acid did not enhance nickel accumulation in either of the two species. Additionally, citric acid application increased the level of impurities in the bio-ores from the plants. Synchrotron X-ray fluorescence analysis revealed that the tissue-level distribution of metals within the plants, with or without citric acid treatment, highlights manganese toxicity as the most significant limitation for Ni phytoextraction. The results of this study show that the use of citric acid is not an effective strategy for nickel agromining; however, its capacity to mobilize multiple metals suggests potential applicability in phytoremediation of multicontaminated soils.

## Graphical abstract

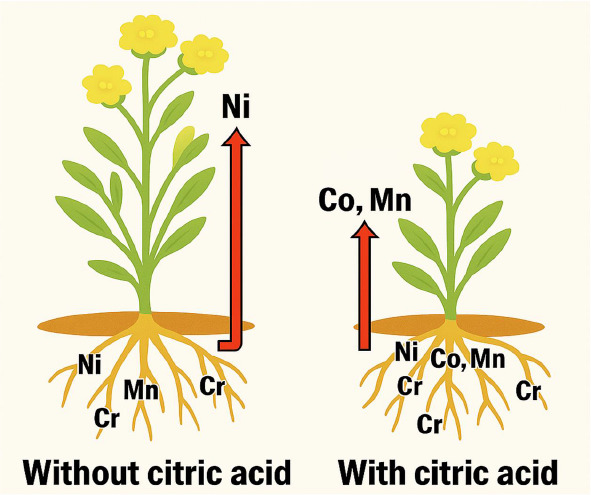

## 1 Introduction

Nickel (Ni) agromining in ultramafic soils represents an environmentally sustainable complement to conventional Ni mining that has garnered significant interest within the scientific community, showing potential for large-scale economic viability.^1–3^ This technique utilizes rare plants known as hyperaccumulators, which can concentrate Ni at levels hundreds to thousands of times higher than most other plant species, enabling its recovery as bio-ores.^4^ Despite their broad distribution, most research has focused on species from temperate climates, such as the Greek endemic hyperaccumulator Bornmuellera emarginata (Boiss.) Rešetnik can attain Ni foliar accumulation of up to 34,400 mg kg^–1^ with some specimens reaching up to 34,890.^5,6^ Among tropical species, the South African plant Berkheya coddii Roessler has demonstrated Ni accumulation as high as 76,000 mg kg^–1^, highlighting its exceptional potential for Ni agromining.^4^

The success of agromining largely depends on the hyperaccumulation capacity of the species, its ability to produce significant biomass within a relatively short period, and the solubility of Ni in the substrate. While not essential to the process, techniques that enhance Ni solubility in the soil and its uptake by plants can significantly improve phytoextraction performance. These techniques include the application of fertilizers, herbicides, pH adjustment, optimizing planting density, microbial inoculation, and application of chelating agents.^7–10^

Chelating agents increase the solubility of metals in the soil by complexing them, mobilizing metals from recalcitrant compartments, and chemically enhancing root absorption.^11,12^ Natural chelating agents like citric acid (C_6_H_8_O_7_) have shown potential for metal phytoextraction. Despite being less effective than synthetic chelators, they are preferred due to their low persistence and short residence time, which reduces environmental risks. Citric acid is a low molecular weight, water-soluble organic acid characterized by its low cost, rapid biodegradability, and natural origin.^13,14^

The use of citric acid with non-hyperaccumulator plants is well-documented in the literature as an effective strategy to increase metal phytoextraction.^15–18^ However, the interaction between citric acid and hyperaccumulator species yielded mixed results regarding Ni accumulation. For instance, studies have shown that citric acid application at 20 mmol kg^–1^ in serpentine soils increased foliar Ni accumulation by up to 55% in Noccaea caerulescens and in Odontarrhena muralis (Nascimento et al.). Conversely, other studies reported reductions of 54–78% in Ni concentrations in roots and leaves of Halimione portulacoides and Berkheya coddii under similar treatments.^19,20^ These contrasting outcomes suggest that the effectiveness of citric acid depends on factors such as plant species, soil conditions, and chelator-metal affinity.

Studies reporting significant increases in foliar Ni concentration in hyperaccumulators treated with citric acid have not yet provided critical information on the net removal of the metal and the final composition of the bio-ore, which are key parameters for evaluating the efficiency of management practices in agromining.^9,21^ We hypothesize that citric acid can negatively affect Ni agromining by reducing the selectivity of metal absorption and increasing competition between Ni and other comobilized metals. To address this gap, this study aimed to assess the effects of citric acid application on metal solubility in tropical and temperate ultramafic soils, as well as its potential role as a management practice for Ni agromining in the hypernickelophore, i.e., Ni > 1% DW species B. coddii and B. emarginata. Additionally, the metal concentration of the bio-ore was evaluated, and tissue-level distribution was elucidated using synchrotron X-ray fluorescence analysis to observe the effects of citric acid application on metal distribution and dynamics within these species.

## 2 Material and Methods

### 2.1 Soil Sampling and Seed Collection

The tropical ultramafic soil was collected from a depth of 0–20 cm in Buenos Aires, northeastern Brazil (07°44′04″S, 35°24′59″W). The ultramafic temperate soil was collected from a depth of 0–20 cm at the Vosges ultramafic outcrop in northeastern France (47°54.5′N, 06°57.5′E). Seeds of B. coddii were sourced from ultramafic soils in the Mpumalanga area of South Africa, while seeds of B. emarginata were obtained from Phthiotis, an ultramafic site in Greece.^10^

### 2.2 Chemical and Physical Soil Analysis

The soils were air-dried and sieved through a 2.0 mm mesh. The chemical and physical attributes analyzed for the temperate soil were pH in water at a ratio of 1:5 (v/v);^22^ exchangeable cations K^+^, Ca^2+^, Mg^2+^, and Al^3+^ were extracted using hexamminecobalt­(III) chloride.^23^ Available phosphorus was extracted using the Olsen method.^24^ Particle size was determined using the pipet method.^25^ The extractable concentrations of Co, Cr, Cu, Mn, Ni, and Zn were extracted using diethylenetriaminepentaacetic acid (DTPA).^26^ The total metal concentration was extracted by digesting 0.5000 g (Ø < 2.0 mm) of the soil in aqua regia.^27^ All the extracts were filtered (Ø < 2.5 μm); the volumes were calibrated to 25 mL with ultrapure water and stored at 4 °C for later analysis. The same attributes were evaluated for the tropical soil following the methodologies described by the Brazilian Agricultural Research Corporation (EMBRAPA).^28^

### 2.3 Citric Acid Application

Four doses of citric acid (0, 20, 40, and 60 mmol kg^–1^)—equivalent to approximately 0, 3.84, 7.68, and 11.53 g of chelating agent per kg of soil, respectively—were applied to triplicates of 200 g of tropical and temperate ultramafic soils to evaluate the effects of the chelating agent on soil properties and to determine the optimal dose for subsequent experiments with hyperaccumulator plants. Subsamples of 10 g were collected 2 days after citric acid application to measure pH and the soluble concentrations of Ni, Co, Cr, Cu, Mn, and Zn in the soils. The citric acid used was a food-grade product, with an approximate cost of 5 USD kg^–1^.

### 2.4 Experimental Design

Seeds of B. emarginata and B. coddii were germinated in a sand + vermiculite mixture (1:1 v/v). After 15 days of germination, the seedlings were transplanted into plastic pots containing 5 kg of ultramafic soil. The pots were not fertilized to simulate the natural growing conditions. The experiment was conducted in a completely randomized design, with treatments including a control (without citric acid) and a treatment with citric acid (20 mmol kg^–1^), each with four replications.

Bornmuellera emarginata plants were cultivated in the temperate ultramafic soil for 45 days in a growth chamber set at 24 °C, 70% relative humidity, and a 16 h photoperiod. Berkheya coddii plants were grown in the tropical ultramafic soil for 90 days in a greenhouse with an average temperature of 32 °C, 80% relative humidity, and a 12 h photoperiod. All pots were weighed daily and irrigated with distilled water to maintain the soil’s water retention capacity at 80%. The dose of 20 mmol kg^–1^ citric acid was applied on the 40th day of cultivation for B. emarginata and on the 85th day of cultivation for B. coddii.

### 2.5 Plant Analysis

#### 2.5.1 Plant Digestion

The plants were collected and separated into roots and shoots. The plant samples were washed with running water and triple-washed with distilled water, dried in an oven at 65 °C, and pulverized in a ball mill. Subsequently, subsamples of 0.05 g of roots and 0.5 g of leaves were digested with HNO_3_ + H_2_O_2_ (1:2) in a mineralizer block (DigiPREP MS) at 120 °C for 3 h, according to the manufacturer’s protocol for metal determination. All the extracts obtained from the digestions were filtered (Ø < 2.0 μm), and the volume was topped up to 25 mL with ultrapure water.

#### 2.5.2 Synchrotron X-ray Microfluorescence Analysis (μXRF)

Following treatment with citric acid, the leaves from both treated and control samples were cleaned by using deionized water and dried with absorbent wipes (Kimwipes, Kimberly-Clark, Dallas, TX, USA). Subsequently, the collected leaves were sectioned in the central region. A section approximately 1 cm in length was made using a sterile scalpel with the cut oriented perpendicular to the central vein. Thereafter, the samples were immersed in an embedding compound (Optimal Cutting Temperature, OCT, VWR, CA95057–838) and rapidly frozen in isopentane cooled with liquid nitrogen. This procedure ensured rapid sample cryofixation, suppressing biological processes while preserving the elemental distribution as close as possible to its natural state. Cross sections of the frozen-hydrated tissues were subsequently obtained with a thickness of 30 μm by using a cryomicrotome (Leica CM1860 UV, Nussloch, Germany). During sectioning, the cryogenic chamber was maintained at a temperature of −20 °C. The obtained sections were deposited on an Ultralene film (SPEX Certiprep, Metuchen, NJ), mounted on a silicon frame, and stored in liquid nitrogen until analysis.

Micro-X-ray fluorescence (μXRF) analysis was conducted at the TARUMÃ end station of the CARNAUBA Coherent X-ray nanoprobe beamline^31^ installed at the Sirius fourth-generation synchrotron radiation source of the LNLS.^32^ Samples were transferred to the experimental setup under liquid nitrogen vapor to ensure preservation of the cold chain. The samples were raster scanned through the X-ray beam under cryogenic conditions maintained by a cryojet, which kept the samples at temperatures between −80 and −100 °C and a relative humidity of approximately 1%. μXRF maps were collected at 9.725 keV in continuous fly scan mode, with a pixel size of 0.5 × 0.5 μm^2^ and a dwell time of 8 ms per pixel to achieve high spatial resolution. The selection of areas for high spatial resolution mapping was guided by low spatial resolution maps, with a pixel size of 2 × 2 μm^2^ and a dwell time of 5 ms per pixel. X-ray fluorescence (XRF) spectra and elemental imaging distribution (controls and treatments) were processed using the software PyMCA 5.8.9 ^33^ developed by the software group of the ESRF.

#### 2.5.3 Obtaining Bio-Ore

The shoot biomass of the species was dried, ground, and incinerated in a muffle furnace at 550 °C for 3 h. After combustion, the bio-ore was removed from the muffle furnace and weighed. To quantify the Ni concentration in the ashes (bio-ore), 0.2 g was transferred to Teflon tubes, where 8.5 mL of HNO_3_ and 1.5 mL of H_2_O_2_ were added for total digestion in a microwave oven model Ethos EZ (Milestone, Sorisole, Italy).^34^

### 2.6 Metal Determination and Quality Control

The concentrations of metals in the soils, plants, and bio-ore were determined by inductively coupled plasma optical emission spectrometry (ICP-OES) (Thermo Fisher iCAP 6300 Duo). The analyses were carried out in triplicate and with blank tests. All the materials used in the analysis were immersed for 24 h in the HNO_3_ solution (10%) and then washed with distilled water. One milliliter of 0.5 mol L^–1^ Lu solution was added to the extracts (Lu as an internal standard to correct for analytical interferents in the determination of metals by ICP-OES). Three samples with certified concentrations of metals in soils and plants were used: one for soil (SRM 2710a—Montana I Soil) and two for plants (SRM 1570a—Spinach Leaves and a check standard from the hyperaccumulator Noccaea caerulescens).

The recoveries of metals in the certified materials were considered satisfactory and ranged from Mn (85–101%), Cu (89–97%), Ni (90–95%), Zn (96–98%), and Co (103%) for plants and Cr (93%), Zn (96%), Mn (98%), Ni (99%), Co (101%), and Cu (102%) for soil. The regression coefficients of the standard curves of the analyzed metals were all >0.999. A medium-concentration standard solution was inserted into the analytical procedure to test the instrument response’s stability and repeatability; the instrument response’s deviation was <5%.

### 2.7 Data Analysis

The data obtained was submitted to univariate statistical methods (mean and standard deviation). The results of the plant biomass and metal accumulation variables were analyzed for homoscedasticity and normal distribution using the Shapiro–Wilk test (*p* > 0.05) and submitted to analysis of variance (ANOVA, *p* ≤ 0.05). Logarithmic transformations were applied when required to satisfy the normality assumptions. Regression analysis, both linear and nonlinear, was used to investigate the relationship between metal solubility in soils and citric acid doses. Model fit was evaluated using *r* ^2^, *p*-values (<0.05), and residual diagnostics. Pearson’s correlation was carried out to assess the relationship between the spatial distributions of metals in the cross sections of the leaves. The potential for natural and assisted phytoextraction was evaluated by the translocation (TF), bioconcentration (BCF), and removal factors of the metals.^29,30^ Graphical and statistical procedures were carried out using OriginPro 2023, PyMca (version 5.8.9), and SISVAR software (version 5.8).

## 3 Results

### 3.1 Natural and Induced Solubility of Metals in Ultramafic Soils

The soluble metal concentrations in the ultramafic soils of Buenos Aires, Brazil, and Vosges, France, followed a similar pattern, with Ni and Mn being the most soluble elements, followed by Cu, Co, Zn, and minimal levels of chromium (Table 1).

**Table 1:** Chemical and Physical Attributes of the Ultramafic Soils Used in This Study^a^

parameter	unit	Brazil (Buenos Aires)	France (Vosges)
pH (H_2_O)	1:2.5	6.6	7.0
Ca	cmol_c_ kg^–1^	6.1	1.6
Mg	cmol_c_ kg^–1^	7.0	16.5
Al	cmol_c_ kg^–1^	<0.02	<0.02
K	cmol_c_ kg^–1^	0.4	0.1
P	g kg^–1^	2.6	0.004
COS	g kg^–1^	9.5	18.6
Ni total	g kg^–1^	1.9	1.0
Cr total	g kg^–1^	5.4	0.7
Co total	mg kg^–1^	191	NA
Cu total	mg kg^–1^	314	13.1
Mn total	g kg^–1^	2.1	1.1
Zn total	mg kg^–1^	200	56.5
Ni DTPA	mg kg^–1^	8.2	42.0
Cr DTPA	mg kg^–1^	<0.2	<0.2
Co DTPA	mg kg^–1^	0.5	1.1
Cu DTPA	mg kg^–1^	3.7	1.0
Mn DTPA	mg kg^–1^	7.7	12.3
Zn DTPA	mg kg^–1^	0.4	1.5
sand	%	29.1	57.2
silt	%	42.6	30.2
clay	%	28.3	12.6

a NA—Not analyzed.

The soils initially exhibited slightly acidic to neutral pH levels (6.6–7.0). However, 2 days after the application of citric acid at varying doses, the pH dropped to highly acidic levels (4.5–5.5). Citric acid application at doses of 20, 40, and 60 mmol kg^–1^ significantly increased the solubility of Ni, Co, Cr, and Mn in the soils compared to the control. Strong positive correlations were found between citric acid application and the solubility of Ni (0.99), Cr (0.98), Co (0.96), Zn (0.93), Cu (0.87), and Mn (0.86) in the soils. The results revealed a consistent pattern across both ultramafic soils, with a progressive increase in soluble metal concentrations as the citric acid doses increased (Figure 1).

**Figure 1:**
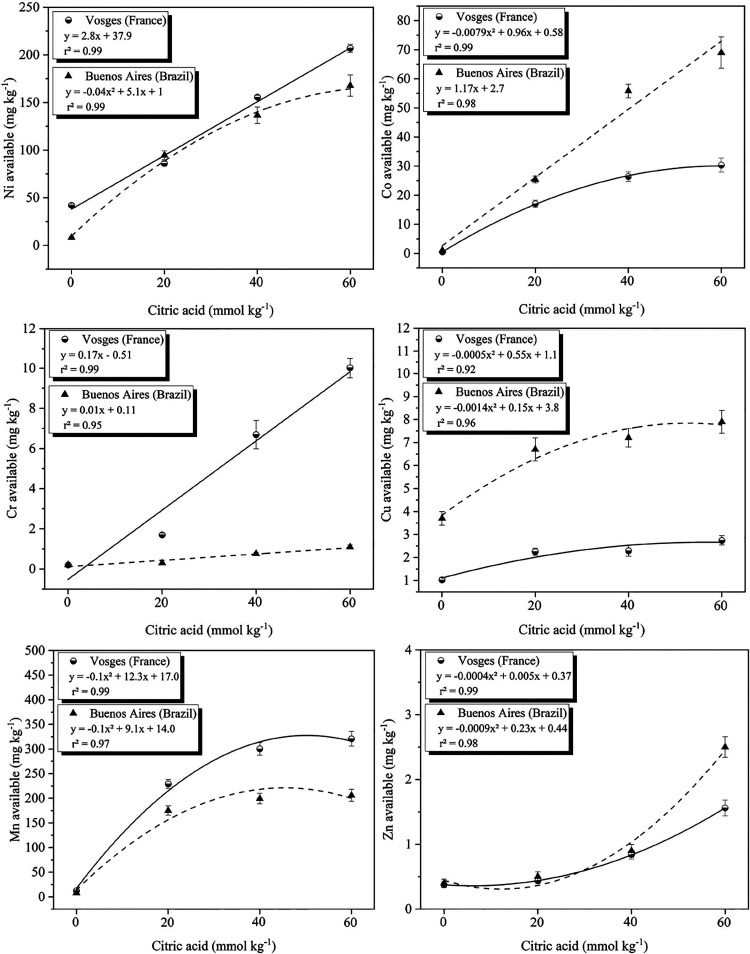
Solubility of Ni, Co, Cr, Cu, Mn, and Zn in the ultramafic soils of Buenos Aires, Brazil, and Vosges, France after application of increasing doses of citric acid (0, 20, 40, and 60 mmol kg^–1^) (ANOVA, *p* ≤ 0.05).

The application of citric acid resulted in notable increases in Ni solubility in the soils: 2–6 times (85–94 mg kg^–1^) for the 20 mmol kg^–1^ dose, 3–16 times (137–155 mg kg^–1^) for the 40 mmol kg^–1^ dose, and 5–20 times (168–207 mg kg^–1^) for the 60 mmol kg^–1^ dose. For Cr, the chelating agent increased its solubility by up to 50 times at the highest dose, although its average solubility remained low (0.5–4.6 mg kg^–1^). The most significant effects of citric acid were observed for Co and Mn, with increases of 18–27 times for Mn (180–321 mg kg^–1^) and 34–168 times for Co (17–69 mg kg^–1^). The significant increase in Ni solubility, and the relatively smaller increases in other metals, was compared to the 40 and 60 mmol kg^–1^ doses. Therefore, a 20 mmol kg^–1^ dose of citric acid was selected for the experiments with the plants.

### 3.2 Effect of Citric Acid on Plant Biomass

The average biomass after 90 days of growth for B. coddii and 45 days for B. emarginata was 5.0 and 4.1 g per plant (shoot) and 1.9 and 0.5 g per plant (root), respectively (Figure 2). The application of citric acid at a dose of 20 mmol kg^–1^ led to a reduction in leaf biomass by an average of 30% for B. coddii and 44% for B. emarginata. Significant reductions in root biomass were also observed, with decreases ranging from 32 to 43% in B. emarginata and B. coddii, respectively.

**Figure 2:**
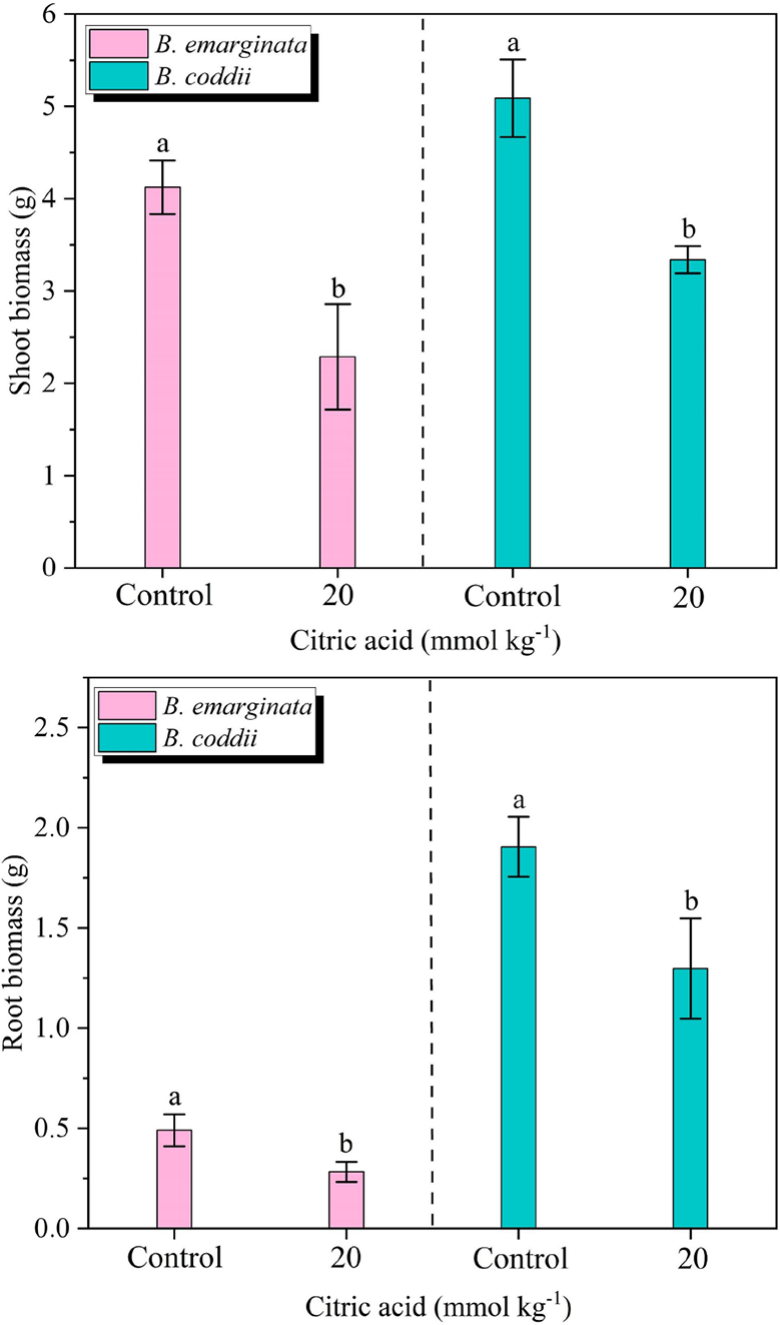
Leaf and root biomass of Bornmuellera emarginata and Berkheya coddii under different citric acid treatments. The differences between treatments were determined by using ANOVA (*p* ≤ 0.05).

### 3.3 Metal Accumulation in B. coddii and B. emarginata

Leaf and root accumulations of all metals in B. coddii were between 2 and 102 times higher than those in B. emarginata, displaying distinct accumulation patterns (Figure 3). The foliar concentrations of Ni were not significantly affected by the application of citric acid, with averages of 4.7 g kg^–1^ in B. emarginata and 14.1 g kg^–1^ in B. coddii. Similarly, root accumulations of Ni did not differ between treatments, with lower concentrations observed in the Greek species B. emarginata (0.6 g kg^–1^) compared to those of the South African B. coddii (5.2 g kg^–1^). Chromium concentrations in B. emarginata were negligible, with an average root accumulation of 4.3 mg kg^–1^ and foliar accumulation below the detection limit (<0.2 mg kg^–1^). In contrast, root Cr concentrations in B. coddii reached 389 mg kg^–1^, and foliar Cr accumulation was significantly affected by citric acid application, increasing from 3 to 50 mg kg^–1^.

**Figure 3:**
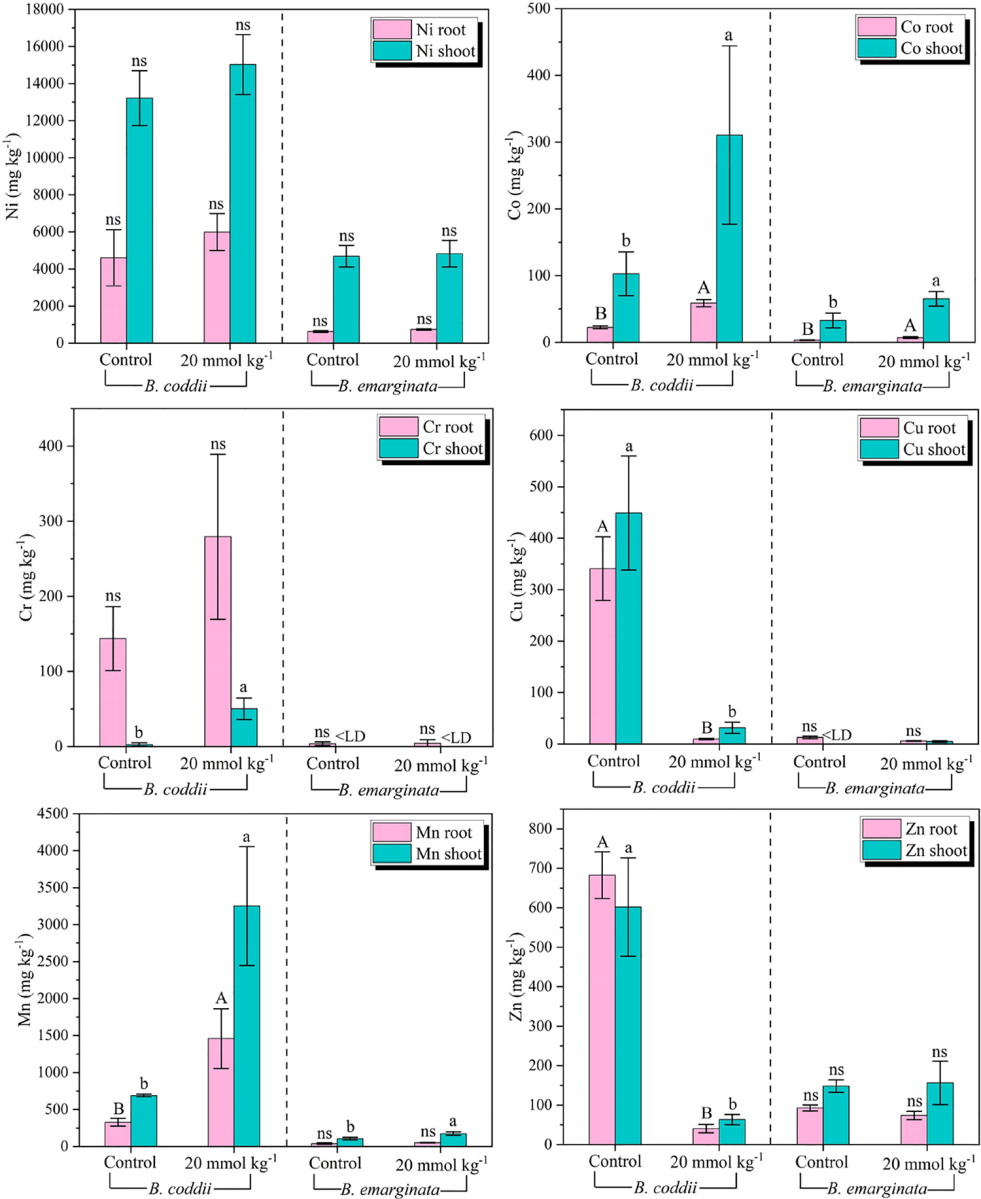
Mean concentration of metals (±standard deviation) in the roots and leaves of Berkheya coddii and Bornmuellera emarginata. LD = limit of detection. ns = nonsignificant. Uppercase letters indicate statistically significant differences in metal concentrations among root samples, and lowercase letters indicate differences among leaf samples under different citric acid treatments (ANOVA, *p* ≤ 0.05).

The application of the organic chelator significantly increased the root and leaf accumulation of Co and Mn in both species. For Co, the leaf concentrations of B. emarginata and B. coddii increased by 90 and 202%, respectively, reaching values between 76 and 444 mg kg^–1^. Root accumulations of Co also increased significantly in both species, ranging from 3 to 59 mg kg^–1^. For Mn, leaf concentrations increased by 64% in B. emarginata and 370% in B. coddii, with the South African species reaching up to 4.0 g kg^–1^. In the roots, the highest Mn accumulation was observed in B. coddii, which reached 1.4 g kg^–1^, four times higher than the control and 31 times higher than the root concentration of B. emarginata. However, the application of the chelator negatively affected the accumulation of Cu and Zn in B. coddii. The leaf Cu concentration decreased from 450 to 31 mg kg^–1^, while the root concentration dropped from 340 to 9 mg kg^–1^. For Zn, the root and leaf accumulations without citric acid were 683 and 602 mg kg^–1^, respectively; however, these values were reduced by 10–17 times after chelator application, resulting in accumulations of 40 and 63 mg kg^–1^, respectively.

### 3.4 Translocation, Bioconcentration, and Removal of Metals

The application of citric acid in the soil (20 mmol kg^–1^) had a significant effect on the factors of bioconcentration (BCF), translocation (TF), and net metal removal by B. coddii and B. emarginata in the ultramafic soils (Table 2).

**Table 2:** Bioconcentration and Translocation Factors and Removal of Metals by B. coddii and B. emarginata before and after Application of Citric Acid (20 mmol kg^–1^) in Ultramafic Soils^a^

translocation factor (TF)							
species	citric acid	Ni	Co	Cr	Cu	Mn	Zn
*B. coddii*	control	2.9 a	4.6 b	0.0	1.3 b	2.1	0.9 b
*B. coddii*	20 mmol kg^–1^	2.5 b	5.3 a	0.2	3.3 a	2.2	1.6 a
*B. emarginata*	control	7.5 a	9.9	0.0	0.0 b	2.7 b	1.6 b
B. emarginata	20 mmol kg^–1^	6.6 b	9.1	0.0	0.8 a	3.3 a	2.1 a

bioconcentration factor (BCF)							
species	citric acid	Ni	Co	Cr	Cu	Mn	Zn
*B. coddii*	control	6.8	0.5 b	0.0005 b	1.4 a	0.3 b	3.0 a
*B. coddii*	20 mmol kg^–1^	7.7	1.6 a	0.009 a	0.1 b	1.5 a	0.3 b
*B. emarginata*	control	4.7	NA	0.0	0.4 a	0.1 b	2.8
B. emarginata	20 mmol kg^–1^	4.8	NA	0.0	0.0 b	0.2 a	2.6

metal removal (mg plant^–1^)							
species	citric acid	Ni	Co	Cr	Cu	Mn	Zn
*B. coddii*	control	67.3 a	0.5 b	0.01 b	2.3 a	2.8 b	3.1 a
*B. coddii*	20 mmol kg^–1^	50.2 b	1.0 a	0.2 a	0.1 b	10.9 a	2.0 b
B. emarginata	control	19.3 a	0.1	0.0	0.0	0.4	0.6 a
*B. emarginata*	20 mmol kg^–1^	11.0 b	0.1	0.0	0.0	0.4	0.3 b

a Translocation factor was calculated as the ratio of Ni in shoots to the total Ni in roots, while the bioconcentration factor was defined as the ratio of Ni concentration in shoots to that in the soil. Means followed by different letters in the same species do not differ by Tukey's test (*p* ≤ 0.05); NA, not analyzed; ns, not significant.

The application of the chelating agent to soil cultivated with B. coddii resulted in a reduction in the translocation factor (TF) for Ni and Cu by 14 and 31%, respectively. Conversely, the TF for Co increased from 4.6 to 5.3, representing a 15% rise, while the TF for Zn increased from 0.9 to 1.6, an increase of 78%. No significant changes were observed in the TFs for Cr and Mn. Regarding the bioconcentration factor (BCF), a substantial increase was recorded for Co, increasing from 0.5 to 1.6, an increase of 220%. In contrast, the BCF for Cu decreased by approximately 93% from 1.4 to 0.1. In terms of net metal removal, applying citric acid reduced Ni removal by 25%, from 67.3 to 50.2 mg plant^–1^, while Cu removal dropped drastically by 96% from 2.3 to 0.1 mg plant^–1^. In contrast, Mn removal increased significantly, increasing from 3.1 to 10.9 mg plant^–1^, a 251% increase.

In B. emarginata, the application of citric acid had more subtle effects. The translocation factor (TF) for Ni decreased from 7.5 to 6.6, representing a 12% reduction, while the TF for Cu increased from 0.0 to 0.8, though the concentrations remained low. There were no significant changes in the TFs for Co, Cr, and Mn. Regarding the bioconcentration factor (BCF), no major changes were observed for the analyzed metals, except for Cu, which decreased from 0.4 to 0. Net Ni removal was reduced by 43%, from 19.3 to 11.0 mg plant^–1^, while Zn removal decreased by 50%, from 0.6 to 0.3 mg plant^–1^. No significant effects were observed on the removal of Co, Cr, and Mn.

### 3.5 Cellular Level Metal Distribution by Synchrotron X-ray Fluorescence Analysis

Nickel, Mn, and potassium (K) were selected to visualize their spatial distribution in the leaf tissues of B. coddii using synchrotron X-ray fluorescence analysis. The elemental maps of B. coddii leaf cross sections revealed no significant differences in the spatial distribution and fluorescence intensity of the elements between treatments, except for Mn. The highest concentrations of Ni were consistently observed near the epidermis and phloem regardless of the treatment (Figure 4a,b). In contrast, Mn was only visualized in the treatment with citric acid, showing a fluorescence intensity five times greater than that of the control. Manganese was predominantly sequestered in the lower epidermis and, to a lesser extent, in the vascular regions (Figure 4c,d). The correlation between Ni and Mn was low in the control treatment (*r* = 0.15) and moderate (*r* = 0.50) following the application of 20 mmol kg^–1^ citric acid.

**Figure 4:**
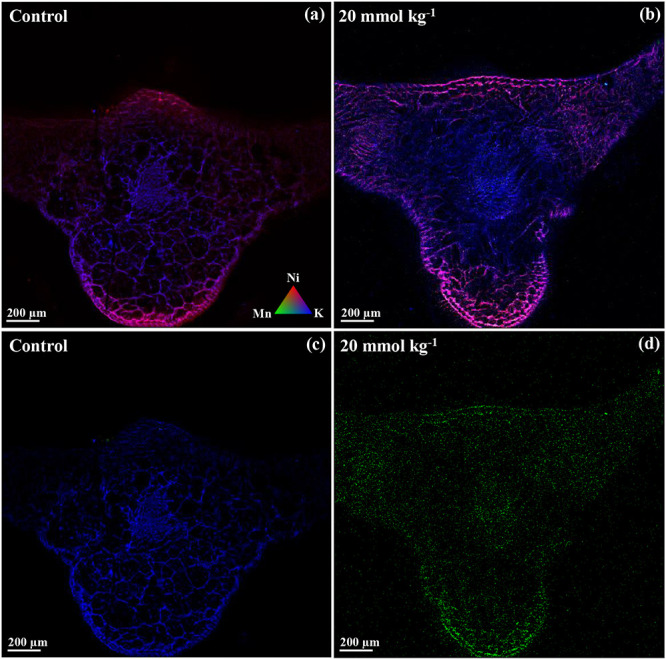
Spatial distribution of Ni (red), Mn (green), and K (blue) in cross sections of frozen-hydrated leaves of Berkheya coddii cultivated in ultramafic soil under two citric acid treatments: 0 (a) and 20 mmol kg^–1^ (b). Images highlight the increase in Mn accumulation after citric acid application, particularly in mesophyll tissues (c and d). The pixel size is 0.5 × 0.5 μm^2^.

### 3.6 Bio-Ore Composition

The leaves of B. emarginata and B. coddii containing between 0.5 and 1.6 wt % Ni were incinerated, and the net weight loss of the vegetal material reached 95%. The metal enrichment factor after incinerating the biomass was 16-fold in B. emarginata and 10-fold in B. coddii, with average Ni concentrations in the ash of 8 wt % for B. emarginata and 16 wt % for B. coddii (Figure 5). The decreasing order of the elemental composition of the bio-ores was for the three most abundant elements: Ca (B. emarginata: 52%, B. coddii: 33%) > Ni (B. coddii: 17%, B. emarginata: 8%) > K (B. coddii: 15%, B. emarginata: 8%).

**Figure 5:**
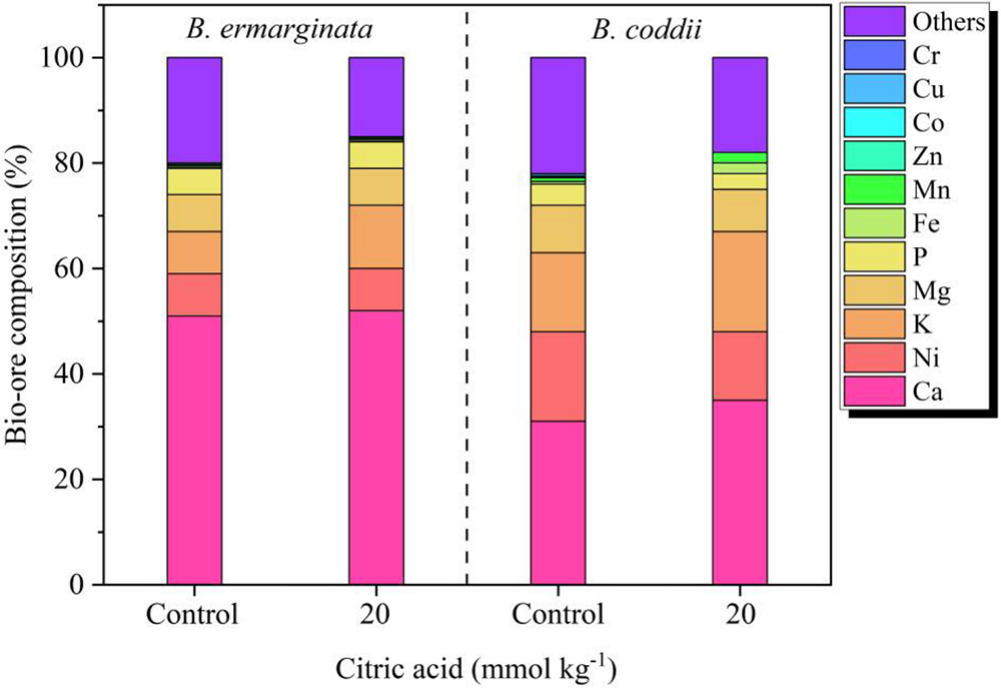
Influence of citric acid on elemental accumulation patterns in the bio-ores of Bornmuellera emarginata and Berkheya coddii, highlighting distinct elemental profiles between species and treatments.

Analysis of the bio-ores under citric acid application revealed significant variations in their elemental composition. Calcium, the predominant element in the bio-ores of both hyperaccumulators, showed no significant changes between the citric acid treatment and the control, indicating that the chelating agent did not affect its absorption in either species. Although B. coddii displayed a clear tendency to accumulate more Ni than B. emarginata, no significant differences in Ni concentration were observed concerning citric acid application. Similar results were found for P, Co, Mg, and Zn, with no notable changes due to the treatment. In contrast, the K concentration in the bio-ores increased with citric acid application, increasing from 8 to 12 wt % in B. coddii and from 15 to 19 wt % in B. emarginata. Overall, citric acid had a more pronounced impact on the bio-ores of B. coddii, leading to increased concentrations of impurities such as Fe, Mn, Cu, and Cr.

## 4 Discussion

In general, the accumulation of elements by plants is directly proportional to their solubility in the soil until it becomes limited by saturation or toxicity.^35^ In ultramafic soils, Ni is predominantly found in recalcitrant fractions, such as Fe oxides, which restrict its immediate availability for plant uptake.^36^ Therefore, strategies to increase Ni solubility in soil are desirable to optimize the metal uptake by plants. Although citric acid application increased Ni solubility by up to 20-fold in the ultramafic soils studied, the chelator action is not selective for Ni alone. These soils also contain high concentrations of elements such as Co, Cr, Mn, and Fe.^37^ An experiment with tropical ultramafic soil incubated with doses of citric acid showed that Co and Cr were mobilized up to 14-fold from the Fe and Mn oxide fractions to the exchangeable fraction when 20 mmol kg^–1^ chelating agent was applied.^18^ This effect is attributed to citric acid’s ability to lower soil pH and form soluble metal complexes, enhancing their solubilization and making them more accessible to plants. Interestingly, our findings contrast with previous results obtained for B. coddii, where citric acid application led to a significant decrease in Ni concentrations.^19^ This discrepancy highlights the critical role of soil conditions, particularly pH and the background concentrations of competing elements, in influencing metal uptake, even when the same species and chelators are used.

The indiscriminate increase in metal solubility can negatively impact the efficiency of Ni agromining, particularly in plant species with a high capacity for specifically accumulating this metal. Additionally, antagonistic interactions between Ni, Mn, Zn, and Fe, due to competition for divalent metal transporters (ZIPs), can limit Ni uptake by plants, thereby restricting the agromining process.^9^ On the other hand, not all metals exhibited the same response to citric acid application in the soil. A reduction in the Cu and Zn concentrations in plant tissues of both species was observed, likely due to competition for absorption sites in the roots, considering that these elements occur at low concentrations in ultramafic soils. Reduction in soil pH caused by citric acid application may also play a role. When citric acid or other natural low-molecular-weight organic acids are applied, it is essential to balance the increase in metal bioavailability with the risk of lowering pH below optimal levels for Ni uptake, which can alter metal speciation, increase competition, and limit Ni translocation to shoots.^18,19^ Irving–Williams series describes the relative stability of divalent transition metal complexes in the order Mn­(II) < Fe­(II) < Co­(II) < Ni­(II) < Cu­(II) > Zn­(II). Accordingly, Cu­(II) forms more stable complexes than Ni­(II), which are in turn more stable than those of Co­(II) and Mn­(II).^51^ This helps explain why citric acid tends to increase the solubility of Mn and Co more effectively than does Ni, potentially limiting Ni enrichment. Thus, when using chelating agents such as citric acid for metal phytoextraction, it is important to consider this stability trend as it influences the relative solubility and plant uptake of target metals.

Regarding the impact of citric acid application on plant biomass, a significant reduction was observed in both leaves and roots, particularly in B. emarginata. This reduction in biomass can be interpreted as a response to stress caused by the increased solubility of trace metals in the soil. A study using citric acid at a dose of 10 mmol kg^–1^ revealed negative effects on root morphology and chlorophyll fluorescence parameters in *Sedum alfredii* Hance.^16^ The increase in the soluble concentrations of metals, such as Co and Mn, although beneficial for absorption, can be toxic to plants, affecting physiological processes such as root growth and photosynthesis. Furthermore, the acidity induced by citric acid application likely exacerbated plant stress, as the shift in soil pH to more acidic conditions (pH 4.5–5.5) limits nutrient absorption and increases exchangeable aluminum. Aluminum toxicity negatively impacts various physiological and metabolic functions, including inducing oxidative damage to biomolecules by elevating reactive oxygen species (ROS) in plant tissues.^38^

Manganese showed the greatest increase in accumulation in the species treated with citric acid in this study, reaching 4000 mg kg^–1^ in B. coddii, a level typical of accumulator species and 20-fold higher than the average found in nonaccumulator plants.^39^ Manganese is essential for plants as it plays critical roles in photosynthesis, particularly in photosystem II, nitrogen metabolism, and the biosynthesis of aromatic amino acids and secondary compounds such as lignin and flavonoids.^40,41^ It also acts as a cofactor for antioxidant enzymes, including manganese superoxide dismutase, as well as enzymes involved in the synthesis of carbohydrates, lipids, and plant resistance to pathogens.^42,43^

However, high Mn can compromise plant health by reducing the activity of antioxidant enzymes, intensifying oxidative stress, and causing cell damage.^44,45^ Its high accumulation can also interfere with the absorption of macronutrients, such as Ca, Mg, P, and K, exacerbating the negative effects on plant growth, development, and biomass production. For example, high Mn concentrations in Phytolacca americana L. have been associated with reduced activities of peroxidase (POD) and ascorbate peroxidase (APX), which may indicate stress responses in plants.^46^ This dual role of Mn as both a nutrient and a potential stress-inducing factor highlights the importance of managing its levels to balance physiological benefits and toxicity risks.

Our results indicate that the application of 20 mmol kg^–1^ citric acid significantly influences the localization and concentration of Mn in areas previously occupied by Ni, reinforcing the limitation of Ni phytoextraction due to competition between these metals. The spatial distribution pattern of Mn in B. coddii, under citric acid treatment, was similar to that described in Grevillea meisneri Montrouz, a known Mn hyperaccumulator.^47^ This similarity suggests that B. coddii, under the conditions of induced phytoextraction, employs a detoxification mechanism akin to that of other hyperaccumulators. Manganese is sequestered in nonphotosynthetic tissues and near the cuticle, minimizing damage to cellular metabolic activities. Additionally, this sequestration strategy provides a defense mechanism against herbivory, further highlighting the adaptive benefits of Mn accumulation under stressful conditions.^47^

Considering the application of the chelating agent on the composition of the bio-ores, the cultivation process with citric acid significantly increased the accumulation of K in the plants and, consequently, in the ash, particularly in B. emarginata. This increase in foliar K concentration can be attributed to the ability of the acid to dissolve potassium feldspars and micas in ultramafic soils, facilitating the uptake of this macronutrient by plants.^48^ Although the release of K is beneficial for the nutritional status of the species, the higher concentration of impurities in the ash complicates the Ni recovery process.^49^ This adds to the cost and increases the likelihood of metal losses during the hydrometallurgical stages, making the process less efficient. Another major limitation of using citric acid in agromining is its high application cost. Even if the lowest tested dose (20 mmol kg^–1^) was effective in enhancing Ni accumulation by hyperaccumulator species, the cost of purchasing and applying the chelating agent would exceed the economic return from extracting over 100 kg of Ni per hectare. This highlights the limited viability of citric acid for agromining purposes and reinforces its more appropriate application in soil decontamination strategies such as phytoextraction.

The differences observed in the accumulation of metals and translocation factors between the two species highlight the importance of considering factors such as the plant’s specificity for each element and the interactions between the chelator and soil attributes. The more pronounced negative effects on B. emarginata compared to B. coddii may be attributed to the ability of B. coddii to hyperaccumulate Co and its higher tolerance to Mn, which mitigates the negative impacts of these elements.^50^

The rare multielement accumulation capacity observed in hyperaccumulators, combined with the average foliar Ni concentration of 1.5 wt % in B. coddii, even when cultivated in soil with relatively low Ni solubility for ultramafic soils (8.2 mg kg^–1^), underscores the species’ strong potential for applications in soil mining or remediation efforts. This unique combination of traits makes B. coddii particularly promising for enhancing the efficiency of agromining practices.

## 5 Conclusions

The results of this study highlight the significant impact of citric acid application on the solubility of trace metals in ultramafic soils and their accumulation in two hypernickelophore species. Citric acid effectively mobilized metals in soils, particularly Ni, Co, and Mn. However, this approach did not enhance Ni agromining, as the increased solubility of Mn competed for absorption sites and altered Ni’s cellular distribution. Additionally, citric acid caused substantial reductions in plant biomass, with losses of up to 44%. These adverse effects limit the feasibility of using citric acid in agromining, as the costs of chelator application, increased impurities in the bio-ore, and lower net Ni removal make the process less economically viable.

While the combination of citric acid with hyperaccumulator plants is not effective for Ni agromining in ultramafic soils, the ability of citric acid to mobilize multiple elements could be valuable in soil remediation contexts involving multielement contamination. In particular, B. coddii exhibited a pronounced response to citric acid application with substantial increases of up to 290% in the net removal of cobalt and manganese.

## #

Botanickel, 54300, Lunéville, France

## References

[1] Ghafoori M., Shariati M., van der Ent A., Baker A. J. M. (2022). Interpopulation
variation in nickel hyperaccumulation and potential for phytomining
by *Odontarrhena penjwinensis* from Western Iran. J. Geochem. Explor..

[2] Nascimento C. W. A., Lima L. H. V., Silva Y. A. J. B., Biondi C. M. (2022). Ultramafic soils
and nickel phytomining opportunities: a review. Rev. Bras. Ciênc. Solo.

[3] Tisserand R., van der Ent A., Nkrumah P. N., Didier S., Sumail S., Morel J., Echevarria G. (2024). Nickel stocks and fluxes in a tropical
agromining ‘metal crop’ farming system in Sabah (Malaysia). Sci. Total Environ..

[4] Reeves R. D., Baker A. J. M., Jafrré T., Erskine P. D., Echevarria G., van der Ent A. (2017). A global database
for plants that hyperaccumulate metal
and metalloid trace elements. New Phytologist.

[5] Reeves R. D., Brooks R. R., Press J. R. (1980). Nickel accumulation by species of *Peltaria* Jacq. (Cruciferae). Taxon.

[6] Jakovljevic K., Bani A., Pavlova D., Konstantinou M., Dimitrakopoulos P. G., Kyrkas D., Reeves R. D., Misljenovic T., Tomovic G., van der Ent A., Baker A. J. M., Baceva A. K., Morel J. L., Echevarria G. (2022). Hyperaccumulator plant discoveries
in the Balkans: Accumulation, distribution, and practical applications. Bot. Serbica.

[7] Bani A., Echevarria G., Sulc E. S., Morel J. L. (2015). Improving the agronomy
of *Alyssum murale* for extensive phytomining: a five-year
field study. Int. J. Phytoremediat..

[8] Bani A., Echevarria G. (2019). Can organic amendments replace chemical
fertilizers
in nickel agromining cropping systems in Albania?. International Journal of Phytoremediation.

[9] Nascimento C. W. A., Hesterberg D., Tappero R. (2020). Effects of exogenous citric acid
on the concentration and spatial distribution of Ni, Zn, Co, Cr, Mn
and Fe in leaves of *Noccaea caerulescens* grown on
a serpentine soil. J. Hazard. Mater..

[10] Ly S. N., Echevarria G., Aarts M. G. M., Ouvrard S., van der
Ent A. (2025). Physiological responses of the nickel hyperaccumulator Bornmuellera
emarginata under varying nickel dose levels and pH in hydroponics. Plant and Soil.

[11] Harmon S. M. (2022). Biodegradable
chelate-assisted phytoextraction of metals from soils and sediments. Curr. Opin. Green Sustainable Chem..

[12] Wang Q., Zhao H., Bekele T. G., Qu B., Chen J. (2023). Citric acid
can enhance the uptake and accumulation of organophosphate esters
(OPEs) in Suaeda salsa rhizosphere: Potential for phytoremediation. Journal of Hazardous Materials.

[13] Freitas E. V. S., Nascimento C. W. A. (2017). Degradability
of natural and synthetic
chelating agents applied to a lead-contaminated soil. Journal of Soils and Sediments.

[14] Shi R., Li T., Wang K., He Y., Fu R., Yu R., Zhao P., Oh K.-C., Jiang Z., Hou J. (2021). Investigation
of the consequences of ultrasound on the physicochemical, emulsification,
and gelatinization characteristics of citric acid–treated whey
protein isolate. Journal of Dairy Science.

[15] Leng Y., Lu M., Li F., Yang B., Hu Z.-T. (2021). Citric acid-assisted
phytoextraction of trace elements in composted municipal sludge by
garden plants. Environ. Pollut..

[16] Li Y., Wang Y., Khan M. A., Luo W., Xiang Z., Xu W., Zhong B., Ma J., Ye Z., Zhu Y., Duan L., Liu D. (2021). Effect of plant extracts
and citric
acid on phytoremediation of metal-contaminated soil. Ecotoxicology and Environmental Safety.

[17] Menhas S., Hayat K., Lin D., Shahid M., Bundschuh J., Zhu S., Hayat S., Liu W. (2024). Citric acid-driven cadmium uptake
and growth promotion mechanisms in *Brassica napus*. Chemosphere.

[18] Nascimento J. S., Lima L. H. V., Biondi C. M., Nascimento C. W. A. (2024). Citric
Acid-Assisted Accumulation of Ni, Cr, and Co by Maize Successively
Grown in a Tropical Ultramafic Soil. Water Air
Soil Pollut..

[19] Robinson B. H., Brooks R. R., Howes A. W., Kirkman J. H., Gregg P. E. H. (1997). The
potential of the high-biomass nickel hyperaccumulator Berkheya coddii
for phytoremediation and phytomining. Journal
of Geochemical Exploration.

[20] Duarte B., Delgado M., Caçador I. (2007). The role of
citric acid in cadmium
and nickel uptake and translocation, in *Halimione portulacoides*. Chemosphere.

[21] Ibrahim E. A. (2023). Effect
of citric acid on phytoextraction potential of Cucurbita pepo, *Lagenaria siceraria*, and *Raphanus sativus* plants exposed to multi-metal stress. Sci.
Rep..

[22] ISO - International Organization for Standardization . Method 10390. Soil, treated biowaste and sludge–Determination of pH. 2021. https://www.iso.org/standard/75243.html.

[23] ISO - International Organization for Standardization . Method 23470. Soil quality — Determination of effective cation exchange capacity (CEC) and exchangeable cations using a hexamminecobalt(III)chloride solution. 2018. https://www.iso.org/standard/68765.html.

[24] ISO - International Organization for Standardization . Method 11263. Soil quality — Determination of phosphorus — Spectrometric determination of phosphorus soluble in sodium hydrogen carbonate solution. 1994. https://www.iso.org/standard/19241.html.

[25] ISO - International Organization for Standardization . Method 1277. Soil quality — Determination of particle size distribution in mineral soil material — Method by sieving and sedimentation. 2020. https://www.iso.org/standard/69496.html.

[26] ISO - International Organization for Standardization . Method 22036. Soil quality — Determination of trace elements in extracts of soil by inductively coupled plasma - atomic emission spectrometry (ICP - AES). 2008. https://www.iso.org/standard/40653.html.

[27] ISO - International Organization for Standardization . Method 11466. Soil quality — Extraction of trace elements soluble in aqua regia. 1995. https://www.iso.org/standard/19418.html.

[28] Teixeira, P. C. ; Donagemma, G. K. ; Fontana, A. ; Teixeira, W. G. Manual de métodos de análise do solo. 2017. http://www.infoteca.cnptia.embrapa.br/infoteca/handle/doc/1085209.

[29] Silva W. R., Silva F. B. V., Araujo P. R. M., Nascimento C. W. A. (2017). Assessing
human health risks and strategies for phytoremediation in soils contaminated
with As, Cd, Pb, and Zn by slag disposal. Ecotoxicol.
Environ. Safety.

[30] Cerdeira-Pérez A., Monterroso C., Rodriguez-Garrido B., Machinet G., Echevarria G., Prieto-Fernandez A., Kidd P. S. (2019). Implementing nickel phytomining in
a serpentine quarry in NW Spain. Journal of
Geochemical Exploration.

[31] Tolentino H. C. N., Gerlades R. R., Da Silva F. M. C., Guaita M. G. D., Camarda C. M., Szostak R., Neckel I., Teixeira V. C., Hesterberg D., Pérez C. A., Galante D., Callefo F., Neto A. C.P., Kofukuda L. M., Sotero A. P.S., Moreno G. B. Z. L., Luiz S. A. L., Bueno C. S. N. C., Lena F. R., Westfahl H. (2023). The CARNAUBA X-ray nanospectroscopy beamline at the
Sirius-LNLS synchrotron light source: Developments, commissioning,
and first science at the TARUMÃ station. J. Electron Spectrosc. Relat. Phenom..

[32] Liu L., Neuenschwander R. T., Rodrigues A. R. D. (2019). Synchrotron
radiation sources in
Brazil. Philosophical Transactions of the Royal
Society A: Mathematical, Physical and Engineering Sciences.

[33] Solé V. A., Papillon E., Cotte M., Walter P., Susini J. (2007). A multiplatform
code for the analysis of energy-dispersive X-ray fluorescence spectra. Spectrochimica Acta Part B: Atomic Spectroscopy.

[34] Zhang X., Houzelot V., Bani A., Morel J. L., Echevarria G., Simonnot M. O. (2014). Selection and combustion
of Ni hyperaccumulators for
the phytomining process. Int. J. Phytoremediat..

[35] Kim R., Yoon J., Kim T., Yang J. E., Owens G., Kim K. (2015). Bioavailability of
heavy metals in soils: definitions and practical
implementation—a critical review. Environmental
Geochemistry and Health.

[36] Lima L. H. V., Silva F. B. V., Silva Y. J. A. B., Veloso V. L., Sousa M. G. F., Sousa Junior V. S., Echevarria G., Nascimento C. W. A. (2024). Integrating environmental, ecological
and human health
risk assessments for heavy metals in tropical ultramafic soils. Sci. Total Environ..

[37] Vithanage M., Kumarathilaka P., Oze C., Karunatilake S., Seneviratne M., Hseu Z., Gunarathne V., Dassanayake M., Ok Y. S., Rinklebe J. (2019). Occurrence and cycling
of trace elements in ultramafic soils and their impacts on human health:
A critical review. Environ. Int..

[38] Parra-Almuna L., Diaz-Cortez A., Ferrol N., Mora M. L. (2018). Aluminium
toxicity
and phosphate deficiency activates antioxidant systems and up-regulates
expression of phosphate transporters gene in ryegrass (*Lolium
perenne* L.) plants. Plant Physiology
and Biochemistry.

[39] Markert B. (1992). Establishing
of “Reference Plant” for inorganic characterization
of different plant species by chemical fingerprinting. Water, Air and Soil Pollution.

[40] Burnell, J. N. The Biochemistry of Manganese in Plants. In Manganese in Soils and Plants. Developments in Plant and Soil Sciences, Graham, R. D. ; Hannam, R. J. ; Uren, N. C. , eds.; Springer: Dordrecht, 1988, Vol. 33.

[41] Alejandro S., Höller S., Meier B., Peiter E. (2020). Manganese in Plants:
From Acquisition to Subcellular Allocation. Frontiers in Plant Science.

[42] Li J., Jia Y., Dong R., Huang R., Liu P., Li X., Wang Z., Liu G., Chen Z. (2019). Advances in the Mechanisms
of Plant Tolerance to Manganese Toxicity. Int.
J. Mol. Sci..

[43] Rai S., Singh P. K., Mankotia S., Swain J., Satbhai S. B. (2021). Iron homeostasis
in plants and its crosstalk with copper, zinc, and manganese. Plant Stress.

[44] Takagi D., Ishiyama K., Suganami M., Ushijima T., Fujii T., Tazoe Y., Kawasaki M., Noguchi K., Makino A. (2021). Manganese
toxicity disrupts indole acetic acid homeostasis and suppresses the
CO_2_ assimilation reaction in rice leaves. Sci. Rep..

[45] Zhang Z., Fu D., Xie D., Wang Z., Zhao Y., Ma X., Huang P., Ju C., Wang C. (2023). CBL1/9–CIPK23–NRAMP1
axis regulates manganese toxicity. New Phytologist.

[46] Zhao H., Wu L., Chai T., Zhang Y., Tan J., Ma S. (2012). The effects
of copper, manganese and zinc on plant growth and elemental accumulation
in the manganese-hyperaccumulator *Phytolacca americana*. Journal of Plant Physiology.

[47] Bihanic C., Petit E., Perrot R., Cases L., Garcia A., Pelissier F., Poullain C., Rivard C., Hossaert-McKey M., McKey D., Grison C. (2021). Manganese distribution in the Mn-hyperaccumulator *Grevillea meisneri* from New Caledonia. Sci. Rep..

[48] Vidal-Torrado P., Macias F., Calvo R., Carvalho S. G., Silva A. C. (2006). Gênese
de solos derivados de rochas ultramáficas serpentinizadas no
sudoeste de Minas Gerais. Revista Brasileira
de Ciência do Solo.

[49] van
der Ent A., Baker A. J. M., Reeves R. D., Chaney R. L., Anderson C. W. N., Meech J. A., Erskine P. D., Simonnot M., Vaughan J., Morel J. L., Echevarria G., Fogliani B., Rongliang Q., Mulligan D. R. (2015). Agromining: farming
for metals in the future?. Environ. Sci. Technol..

[50] Rue M., Paul A. L. D., Echevarria G., van der Ent A., Simonnot M., Morel J. L. (2015). Uptake, translocation
and accumulation
of nickel and cobalt in Berkheya coddii, a ‘metal crop’
from South Africa. Metallomics.

[51] Leussing D. (1960). The estimation
of the stabilities of bivalent transition metal complexes and deviations
from the Irving-Williams order. Talanta.

